# Effect of IVIG therapy on pregnant women with unexplained recurrent spontaneous abortion: a systematic review and meta-analysis

**DOI:** 10.3389/fendo.2024.1381461

**Published:** 2024-08-14

**Authors:** Qiao Ling, Jinfeng Xu, Yuan Tian, Daijuan Chen, Chunheng Mo, Bing Peng

**Affiliations:** ^1^ Department of Obstetrics and Gynecology, West China Second University Hospital, Sichuan University, Chengdu, China; ^2^ West China School of Medicine, Sichuan University, Chengdu, China; ^3^ Key Laboratory of Birth Defects and Related Diseases of Women and Children of MOE, West China Second University Hospital, Sichuan University, Chengdu, China

**Keywords:** intravenous immunoglobulin, unexplained recurrent spontaneous abortion, antiphospholipid syndrome, treatment, meta-analysis

## Abstract

**Objective:**

To assess the effect of intravenous immunoglobulin (IVIG) therapy on unexplained recurrent spontaneous abortion (URSA).

**Methods:**

We retrieved all randomized controlled trials (RCTs) related to the effect of IVIG therapy on URSA in the following databases: PubMed, Embase, Web of Science, and Cochrane Central Register of Controlled Trials before April 30, 2023, according to the PRISMA statement. The therapeutic effect of IVIG was measured by live birth rates. Quality assessment was conducted independently by two reviewers, based on the Newcastle‐Ottawa scale. For the meta-analysis, we used odds ratios (random effects model and fixed effects model). The between-study heterogeneity was assessed by the Q test. Publication bias was assessed by funnel plots.

**Results:**

A total of 12 studies with 751 participants were included in this meta-analysis. There was no statistical significance [OR = 1.07, 95%CI (0.65, 1.75), *P*=0.80] between the IVIG group and the non-IVIG group, including low molecular weight heparin (LMWH) plus low-dose aspirin (LDA), intralipid, multivitamins, albumin, and normal saline. A subgroup analysis was conducted according to the different treatment regimens of the non-IVIG group. Compared to the placebo group, including multivitamins, albumin, and saline, the live birth rate of the IVIG group is superior, but there was no statistical significance [OR =1.43, 95%CI (0.99, 2.07), *P*=0.05]. Another subgroup analysis was performed according to URSA with positive for antiphospholipid antibodies (aPLs). Results showed the live birth rate of IVIG on URSA with positive for aPLs is inferior to that of LMWH plus LDA [OR = 0.25, 95%CI (0.11, 0.55), *P*=0.0007].

**Conclusions:**

IVIG didn’t increase the live birth rate of URSA compared to placebo. Conversely, compared with the IVIG, the LMWH plus LDA treatment schedule can increase the live birth rate of URSA with positive for aPLs.

## 1 Introduction

As reported, the prevalence of recurrent spontaneous abortion (RSA) is 1%-5% (1) and is increasing year by year (2). What’s more, RSA affects around 1%-2% of couples with fertility needs (3). However, the definition of RSA is still debated, with the main points of contention being the number of miscarriages and gestational age (4). The British College of Obstetricians and Gynaecologists (RCOG) defines RSA as three or more spontaneous abortions before the 24^th^ week of gestation (5). The American Society for Reproductive Medicine criteria (ASRM) defines RSA as two or more spontaneous abortions, without limiting the gestational age (6). The European Society of Human Reproduction and Embryology (ESHRE) Guideline Group on RPL defines RSA as two or more miscarriages before the 24^th^ week of gestation (3). And, it is estimated that nearly 5% of women will experience two consecutive miscarriages, and only 1% experience three or more times (7). Etiological screening of RSA patients with two previous miscarriages and those with three or more previous miscarriages found no significant difference, which supports the inclusion of patients with two or more prior miscarriages in RSA management (8). Therefore, in our meta-analysis, RSA is defined as two or more consecutive miscarriages before the 24^th^ week of gestation.

The etiology of RSA (7) is complex, including chromosomal or genetic abnormalities, anatomical abnormalities, autoimmune diseases, the prethrombotic state (PTS), endocrine system disorders, infectious causes, male causes, environmental psychological causes, and so on. However, a large proportion of RSA patients (approximately 40%-75%) have no known cause, known as the unexplained RSA (URSA) (5, 7). According to reports, 8%-46% of patients with RSA have positive for antiphospholipid antibodies (aPLs) (7). It is worth emphasizing to URSA patients that the chance of a successful pregnancy in the future may exceed 50% to 60% (7). Therefore, exploring an effective treatment for URSA may greatly improve the live birth rate of RSA patients.

It is recorded that the occurrence of URSA is related to the imbalance of maternal-fetal immunity (5, 9). In recent years, many treatments for maternal-fetal immune imbalance have emerged in clinical practice. For example, lymphocyte immunotherapy (LIT), intravenous immunoglobulin (IVIG), tumor necrosis factor α (TNF-α), granulocyte colony-stimulating factor (G-CSF), intralipid therapy, progesterone therapy, anticoagulation therapy, immune-suppressant (mainly including glucocorticoids and cyclosporine A), traditional Chinese medicine therapy (10), low molecular weight heparin (LMWH) plus low-dose aspirin (LDA) (11) and so on. However, most of these treatments are laboratory-based, and their efficacy and safety in clinical are still controversial.

Accumulating evidence suggests that, *in vitro* and *in vivo* models, IVIG plays a potential role in immune modulation and inflammation regulation by upregulation of Receptor I for the Fc Region of Immunoglobulin G (FcγRI) and Receptor III for the Fc Region of Immunoglobulin G (FcγRIII) with downregulation of Receptor II B for the Fc Region of Immunoglobulin G (FcγRIIB) receptors, neutralizing autoantibodies, triggering the expansion of the regulatory T (Treg) cells, and reducing natural killer (NK) cell levels and activity (12). At present, there are also some updates on the mechanism of IVIG in URSA with immune imbalance. The possible mechanisms of IVIG preventing URSA are as follows (11–13): down-regulating the function of B cells, inhibiting the anti-idiotypic effect of autoantibody, reducing the phagocytosis induced by Fc receptor, increasing the regulation of T cells, reducing the complement activation system, and inhibiting the expression and function of cytokines. However, the exact mechanism of IVIG’s effect on URSA has not yet been fully investigated.

To date, numerous clinical trials have been conducted to explore the efficacy of IVIG for URSA. A double-blind randomized trial (14) in 1994 indicated there was no conclusive evidence that IVIG could prevent further miscarriage in women with URSA. However, a randomized controlled trial (RCT) (15) conducted by Hideto Yamada in 2022 showed that IVIG increased ongoing pregnancy and live birth rates in 50 patients with URSA who had 4 or more miscarriages. The effect of IVIG on URSA is a lack of consistency. Therefore, this meta-analysis aims to evaluate the effect of IVIG on URSA by synthesizing all randomized controlled trials (RCTs) published on April 30, 2023.

## 2 Materials and methods

### 2.1 Data source and search strategy

Two researchers separately retrieved the following databases: PubMed, Embase, Web of Science, and Cochrane Central Register of Controlled Trials. Combinations of MeSH terms, “Abortion, Habitual”, “Recurrent abortion”, “Immunoglobulin” and “Immunoglobulins, Intravenous” and their entry terms were used. Queries were limited to human studies. The systematic search strategy is outlined in Appendix S1. Publications dated before April 30, 2023.

### 2.2 Study selection

Two researchers respectively screened the full text, the title, and the abstract of the search results. First of all, we removed the repeated articles. Then, we respectively selected articles based on the following inclusion and exclusion criteria. As for debate articles, we discussed with the third researcher to get a result. Two reviewers (Q.L. and J.F.X.) independently evaluated the titles and abstracts. Duplications were removed using ENDNOTE online software and manually. Disagreements were resolved by discussion among authors; if required, a third investigator (B.P.) was involved to resolve the disagreement between evaluators.

The inclusion criteria were: 1) two or more URSA before the 24^th^ week of gestation; 2) intervention groups only received IVIG; 3) no IVIG in control groups; 4) outcomes of the trials included live birth rate; 4) RCTs. The exclusion criteria were: 1) duplicated articles; 2) Case series, case reports, book chapters, review articles, letters to editors, conference reports, cross-sectional studies, case-control studies, cohort studies, and observational prospective studies; 3) full manuscripts not accessible; 4) non-human studies; 5) participants with infectious, genetic, endocrine or anatomical abnormality; 6) intervention group received a combination of IVIG and another drug or no IVIG; 7) the outcome wasn’t live birth rate; 8) non-English language.

### 2.3 Outcomes

The outcome was the number of live births or the live birth rate.

### 2.4 Data Extraction and Risk of Bias Assessment

Two researchers respectively extracted baseline information from every included study, which included the first author, year of publication, inclusion criteria of participants, intervention therapeutic regimen, study design, and the live birth rate as treatment outcome.

We used the risk of bias assessment tables from the Cochrane RevMan software of Cochrane Centre (5.4.7) to evaluate the risk of bias in the included studies. Two researchers respectively evaluate the methodological quality of each included RCTs.

### 2.5 Data Synthesis

We followed the MOOSE checklist and PRISMA guidelines for this systematic review (16, 17). We used odds ratio (OR) and 95% confidence interval (95%CI) to calculate binary data outcomes. Data analysis was conducted using Cochrane RevMan software of Cochrane Centre (5.4.7). The heterogeneity of the meta-analysis was assessed by the chi-squared method. The random-effect model was used in case of significant heterogeneity among studies (*P* ≥0.05, *I^2^* >50%). Subgroup analysis was conducted to assess the potential sources of heterogeneity. Forest plots were used to represent the statistical data graphically. Publication bias was assessed by funnel plots. When a funnel chart is symmetric, then publication bias is less likely to exist and vice versa.

## 3 Results

### 3.1 Study selection

**Figure 1:**
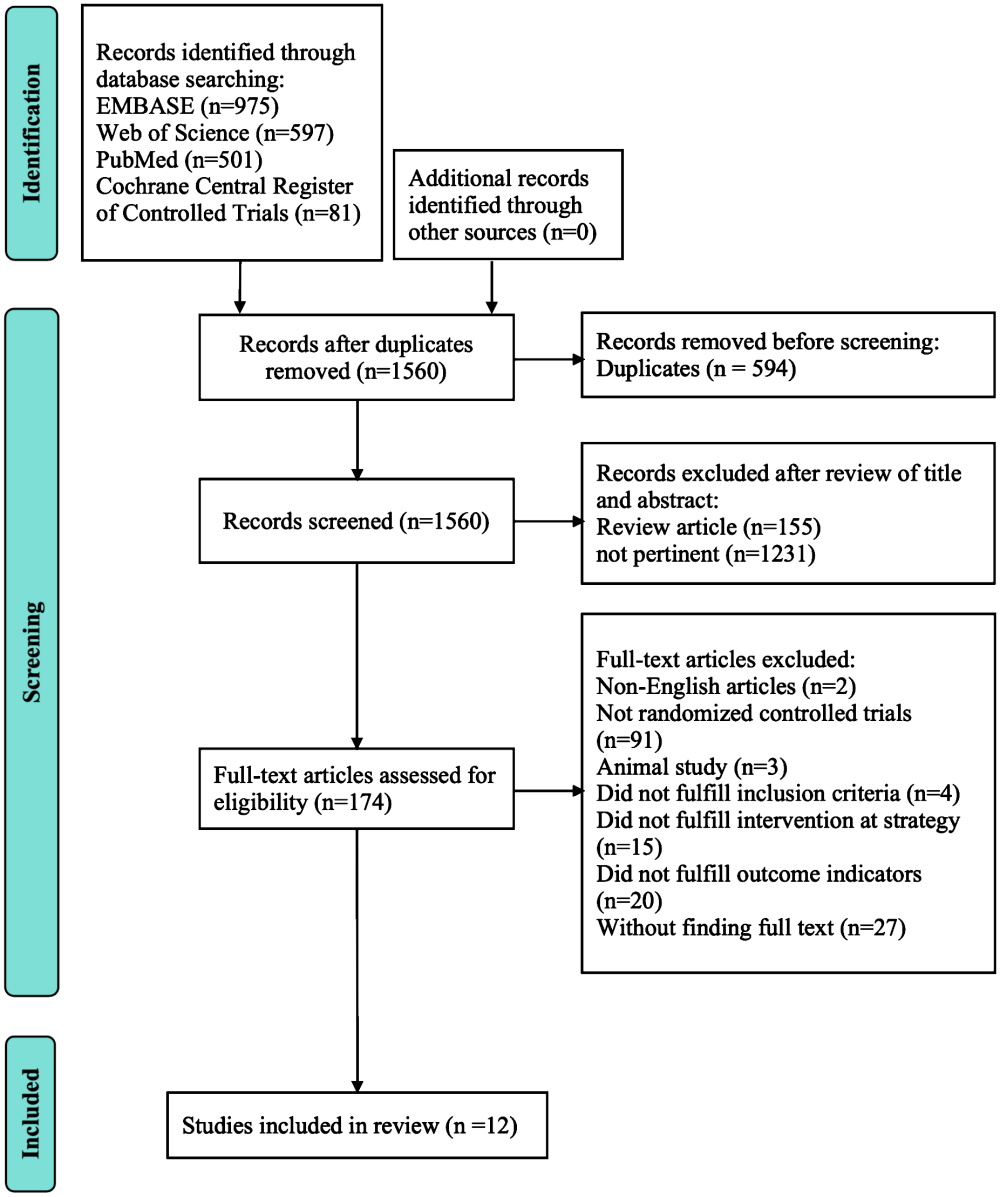
PRISMA flow diagram of systematic literature search.

A primary retrieval found 2154 relevant articles, including 975 articles from Embase, 597 articles from the Web of Science, 501 articles from PubMed, and 81 articles from Cochrane Central Register of Controlled Trials. Firstly, we excluded 594 duplicated articles. Then, we excluded 155 review articles and 1231 unrelated articles by reviewing the title and abstract. After that, we respectively carefully perused the remaining 174 articles in full text. Twelve RCTs (14, 15, 18–27) with 751 patients were selected for detailed assessment. **Table 1** depicts the characteristics of the included studies. **Figure 1** depicts the review flow diagram.

**Table 1:** The characteristics of the included studies.

Study	Study design	Participants (inclusion criteria)	Therapeutic strategy	Time of administration	Total number of achieved pregnancies	IVIG group			Non-IVIG group		
						intervention	Number of live births	Number of achieved pregnancies	intervention	Number of live births	Number of achieved pregnancies
the German RSA/IVIG group 1994 (13)	Multi-centre randomized double-blind	URSA (N≥3) < 16^th^ GW and no live birth	30 g of IVIG (600ml) or 5% albumin (600ml) initiated at <8th GW of gestation and 20g every 3 weeks until 25th GW.	pregnancy	64	IVIG	20	33	ALB	21	31
Coulam CB 1995 (18)	Multi-centre randomized double-blind	URSA (N≥2) < 20^th^ GW	500 mg/kg/month IVIG versus 0.5% albumin was given during the follicular phase and every 28 days until pregnancy was achieved and then continued until 28th–32th GW.	pre-pregnancy + pregnancy	61	IVIG	18	29	ALB	11	32
Perino A 1997 (23)	Multi-centre randomized double-blind	URSA (N≥3) < 12^th^ GW and no live birth	IVIG versus 5% human albumin was given two initial doses of 25 g/day on 2 consecutive days in five 100 ml vials and a third dose of 25 g was administered 3 weeks later when ultrasound scanning confirmed an ongoing pregnancy.	pre-pregnancy + pregnancy	46	IVIG	16	22	ALB	20	24
Stephenson MD 1998 (24)	Randomized double-blind	URSA (N≥2) < 20^th^ GW	500 mg/kg IVIG or normal saline was given initially at a rate of 60 ml/h and gradually increased to 180 ml/h in the follicular phase of the subject’s menstrual cycle. During pregnancy, the participant received the same infusion every 4 weeks until the 18th GW.	pre-pregnancy + pregnancy	41	IVIG	12	20	NS	10	21
Jablonowska B 1999 (20)	Multi-centre randomized double-blind	URSA (N≥2) < 20^th^ GW	20 g of IVIG (400 ml) or saline (400 ml) every 3 weeks on five occasions during pregnancy.	pregnancy	41	IVIG	17	22	NS	15	19
Mahmoud F 2004 (21)	Randomized double-blind	URSA (N≥3) 6th ~ 22^nd^ GW and positive for APS	500 mg/kg/month IVIG or multivitamins was given from confirming pregnancy to about 34th GW. The regimen was 0.5 mg/kg body weight intravenously daily for 5 days every month.	pregnancy	15	IVIG	5	7	multivitamins	6	8
Stephenson MD 2010 (25)	Multi-centre randomized double-blind	URSA (N≥3) ≤20^th^ GW and ≥1 time of live birth	500 mg/kg IVIG or normal saline was administered 14–21 days from the projected next menstrual period. The infusion rate was 60 ml/h for the first hour, then increased to a maximum of 180 ml/h. During pregnancy, the participant received the same infusion every 4 weeks until the 18th–20th GW.	pre-pregnancy + pregnancy	47	IVIG	16	23	NS	15	24
Christiansen OB 2015 (17)	Multi-centre randomized double-blind	URSA (N≥3) < 14^th^ GW and be fathered by the present partner OR RSA (N≥4) < 14^th^ GW and ≥1 time of live birth > 28^th^ GW	IVIG or human albumin at each infusion, participants weighing <75 kg before pregnancy were given 24 g (200ml), and for those weighing ≥75 kg, 36 g (300ml). The second infusion was given 3–6 days after the first and subsequently, three infusions were given at intervals of 6–8 days and three infusions at intervals of 12–16 days (Figure 1). Thus in ongoing pregnancies, a total of eight infusions were given until 14th–15th GW. Every infusion was given over 3–4 hours.	pregnancy	82	IVIG	23	42	ALB	20	40
Yamada H 2022 (14)	Multi-centre randomized double-blind	URSA (N≥4) < 22^nd^ GW and no live birth	IVIG of 400 mg/kg or NS of 8 mL/kg was administered by intravenous drip infusion for five consecutive days. Treatment was initiated at 4 to 6 weeks and 6 days of gestation after the gestational sac was identified by ultrasonography.	pregnancy	99	IVIG	29	50	NS	17	49
Meng LL 2016 (22)	Randomized	URSA (N≥3) < 12^th^ GW	Intralipid Group: The first intravenous injection of 20 % intralipid (250 mL) was administered on the third day of the menstrual cycle. The injection time was no less than 2 hours. Subsequently, repeated injections (250 mL) were given every 2 weeks before pregnancy and once a week after pregnancy until the 12th GW. Immunoglobulin Group: 25 g immunoglobulin was given to the patients on the 8th, 9th, or 10th day of the menstrual cycle. The injection time was no less than 8 h. Subsequently, repeated injections (25 g) were given every month before pregnancy and once a week after pregnancy until the 12th GW.	pre-pregnancy + pregnancy	137	IVIG	48	67	intralipid	39	70
Triolo G 2003 (26)	Randomized double-blind	URSA (N≥3) < 10^th^ GW and 2 times of positive results for aCL IgG①	IVIG Group: IVIG was given from confirming pregnancy to 31st GW or at the time of miscarriage. The dosage of IVIG was 400 mg/kg/day given for 2 consecutive days followed by a single dose each month. LDA+LMWH Group: LDA (75 mg/day) and LMWH (self-administered injection; 5700 IU/day) started at the time of confirming pregnancy, and LDA ended at 34th GW(LDA) and LMWH ended at 37th GW or at the time of miscarriage.	pregnancy	40	IVIG	12	21	LDA+LMWH	16	19
Dendrinos S 2009 (19)	Randomized	URSA (N≥3) < 10^th^ GW and positive aPLs antibodies②	IVIG Group: IVIG (400 mg/kg every 28 days) was given from confirming pregnancy to 32th GW. LDA+LMWH Group: LDA (75 mg/day) and LMWH (4500 IU/day) started at the time of confirming pregnancy, and LDA ended at 32nd GW(LDA) and LMWH ended at 38th GW or at the time of miscarriage.	pregnancy	78	IVIG	15	38	LDA+LMWH	29	40

Note: ① positive results for aCL IgG: the levels of IgG phospholipid units (GPL) 40 with testing performed at intervals of 3 months; ② positive aPLs antibodies: anticardiolipin antibody of IgG and/or IgM isotype in blood, present in medium or high titer, or on 2 or more occasions at least 6 weeks apart; and lupus anticoagulant present in plasma, on 2 or more occasions at least 6 weeks apart; ③ the abbreviations: URSA - unexplained recurrent spontaneous abortion; N - the number; GW - gestational week; APS - antiphosphollipid antibody syndrome; IVIG - intravenous immunoglobulin; ALB - albumin; NS - normal saline; LDA - low-dose aspirin; LMWH - low molecular weight heparin.

### 3.2 Study characteristics and Quality assessment

**Figure 2:**
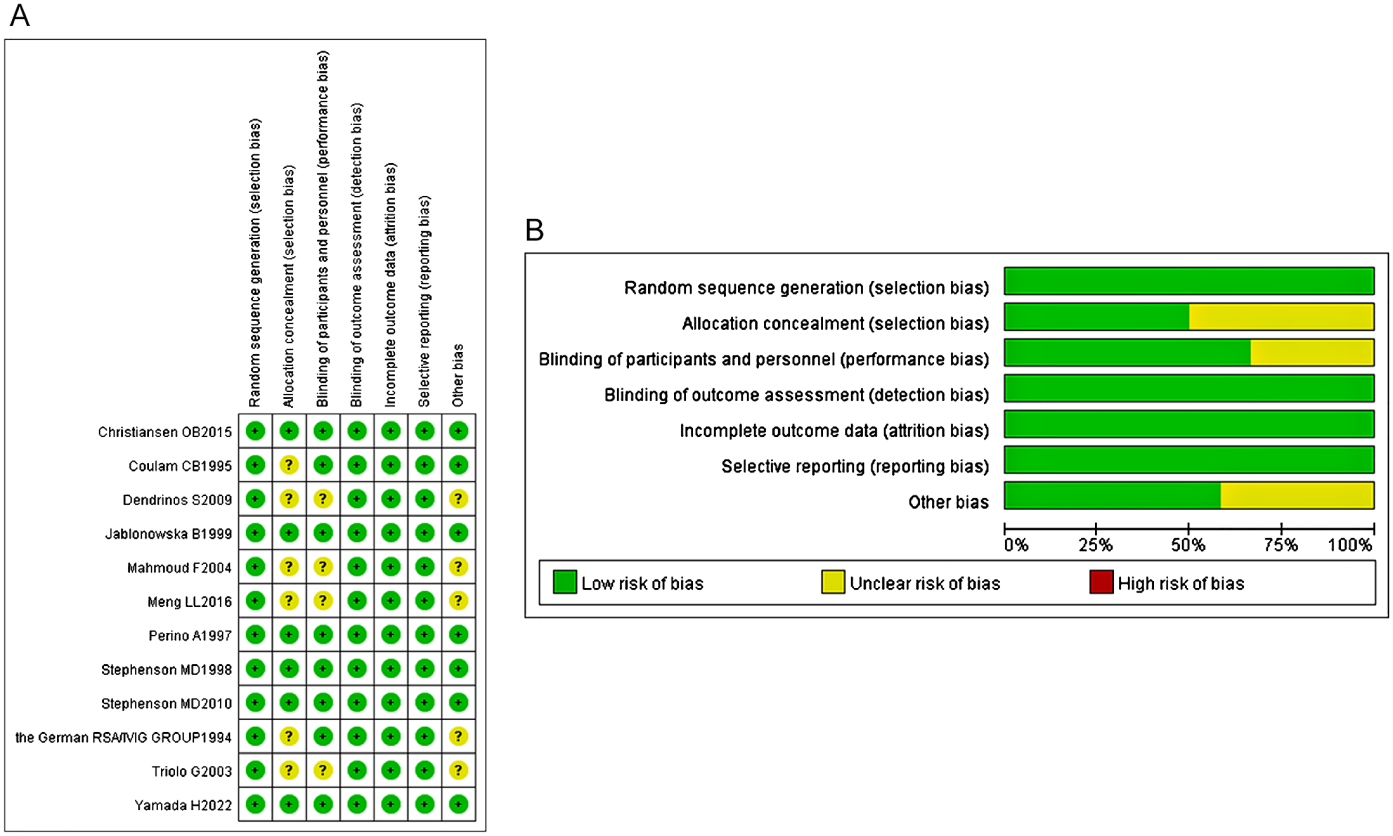
**(A)** The risk of bias assessment of each included study. **(B)** The risk of bias assessment of each included study.

Using the risk of bias assessment tool to evaluate the methodology of each included trial. Overall, all the included studies (12 articles) were lowly risky, but 6 studies had an uncertain risk of biases in the following aspects: random sequence generation, allocation concealment, blinding of participants and intervention providers, and other unknown source of bias (**Figures 2A, B**).

### 3.3 Effect of IVIG on URSA

#### 3.3.1 Live birth rate between the IVIG group and the non-IVIG group

**Figure 3:**
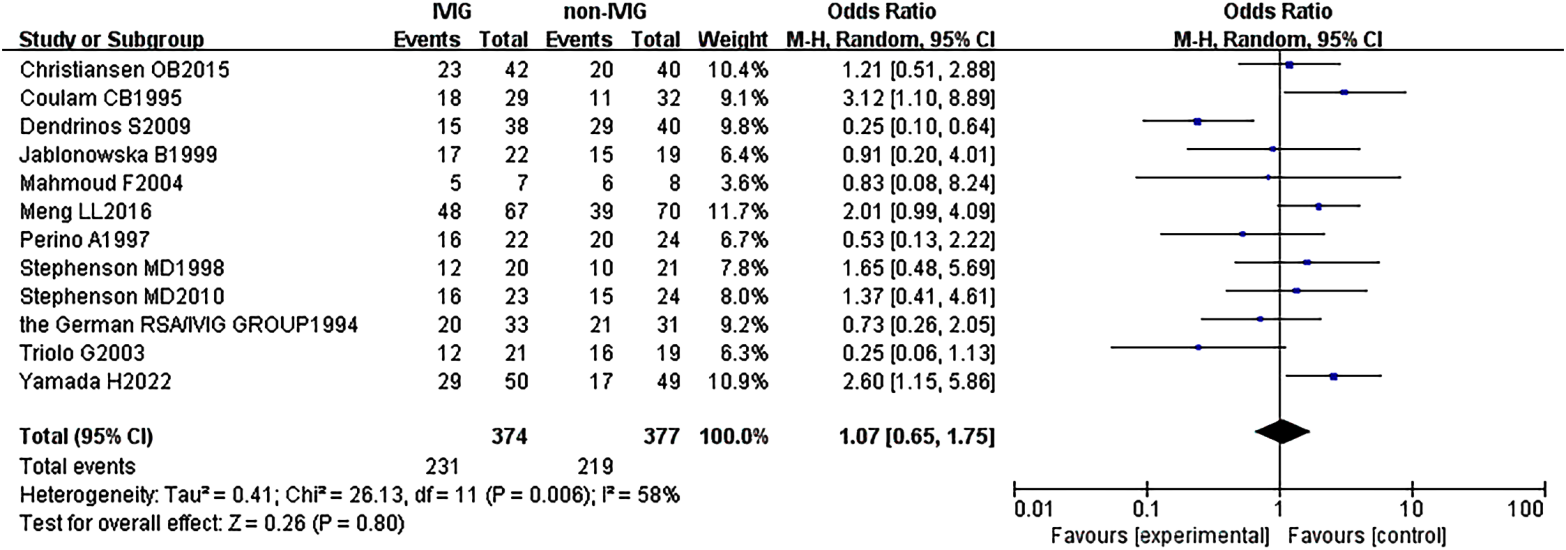
Live birth rate between the IVIG group and the non-IVIG group in patients with recurrent spontaneous abortion.

**Figure 4:**
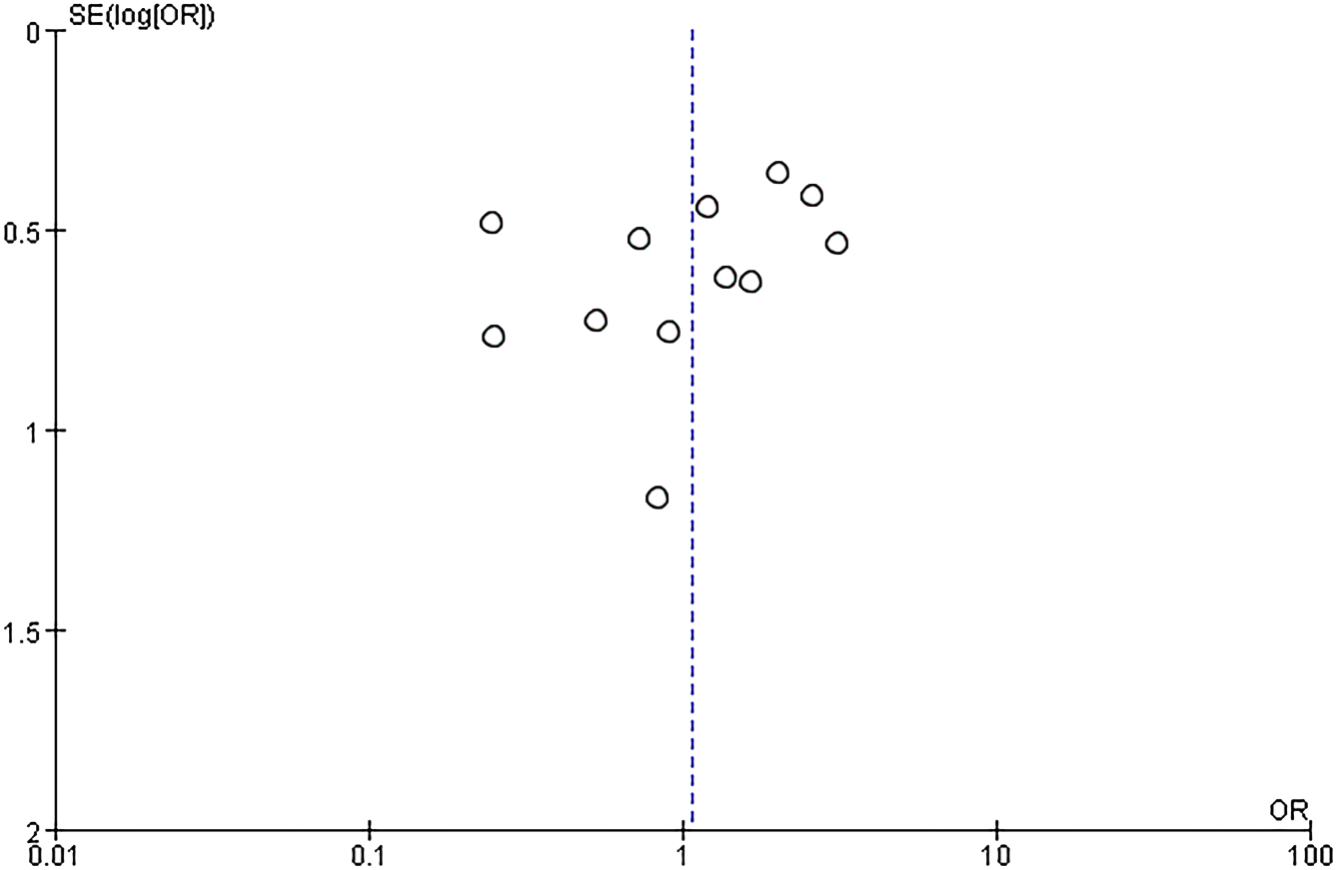
Funnel plot assessment of publication biases (OR, Odds Ratio; SE, standard error).

Twelve RCTs (751 patients) were included, with 374 IVIG users and 377 non-IVIG users. The non-IVIG group refers to the no use of IVIG at the time of treatment, including low molecular weight heparin (LMWH) plus low-dose aspirin (LDA), intralipid, multivitamins, albumin, and normal saline in this meta-analysis. Some heterogeneity was detected among the studies (*P* = 0.006, *I* ^2^ = 58%). Thus, the random effects model was used to conduct data analysis. Results showed that the live birth rate of the IVIG group is higher in comparison with the non-IVIG group, but there was no statistical significance [OR = 1.07, 95%CI (0.65, 1.68), *P*=1.75] (**Figure 3**). All plots fall in the funnel figure and are almost symmetric (**Figure 4**). Therefore, no significant publication bias was identified.

#### 3.3.2 Live birth rate between the IVIG group and the placebo group

**Figure 5:**
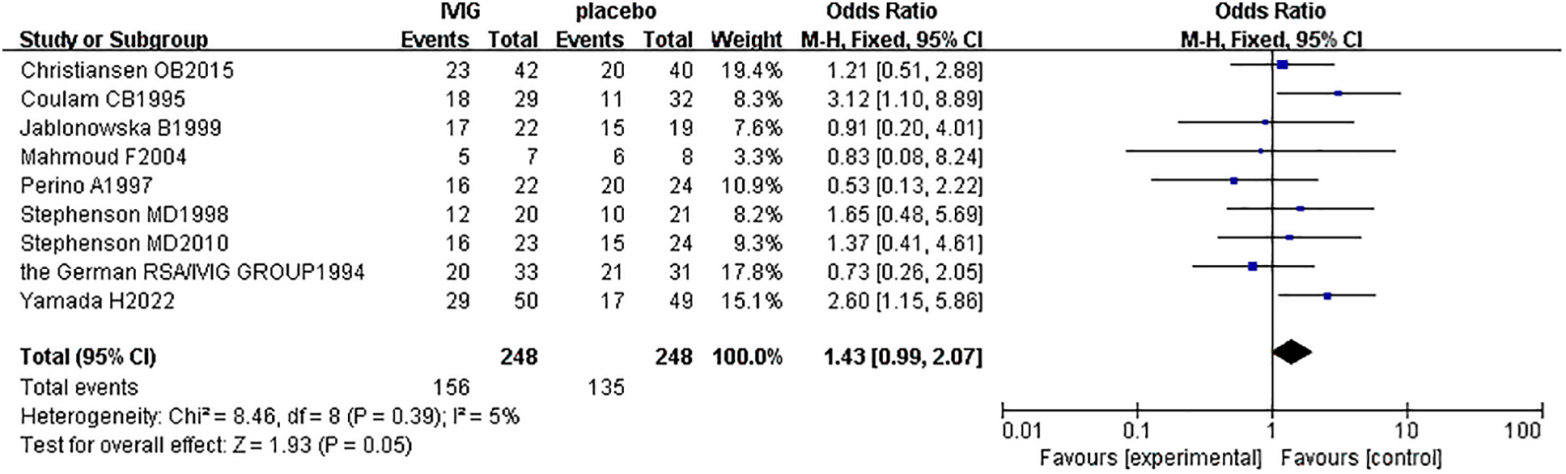
Live birth rate between the IVIG group and the placebo group in patients with recurrent spontaneous abortion.

**Figure 6:**
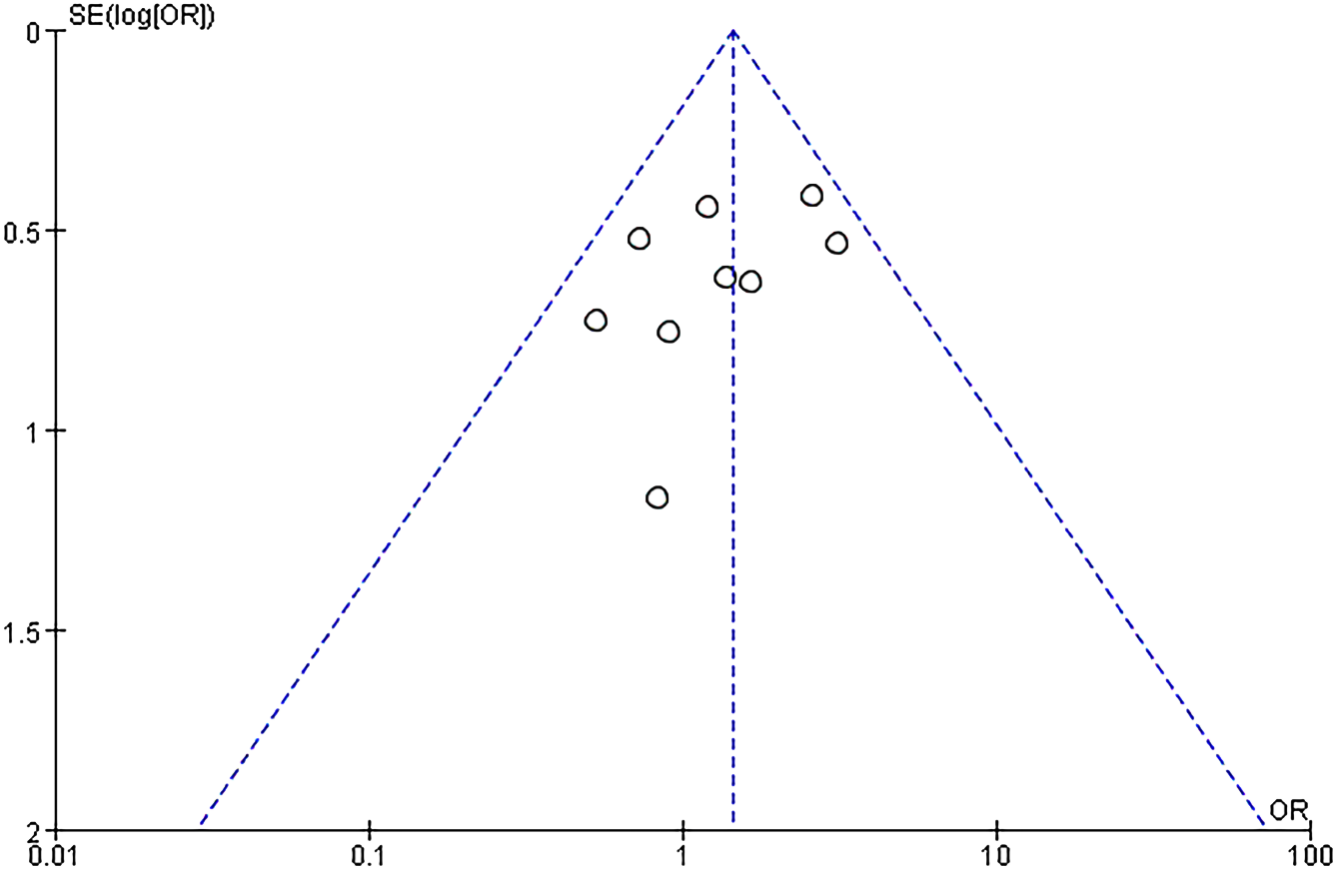
Funnel plot assessment of publication biases (OR, Odds Ratio; SE, standard error).

A subgroup analysis was conducted according to the different treatment regimens of the non-IVIG group. Placebo group refers to the use of no-effect reagents on URSA at the time of treatment, including multivitamins, albumin, and saline in this meta-analysis. Nine studies with 496 patients reported the live birth rate of IVIG therapy (248 patients) and placebo group (248 patients). There was no heterogeneity among the studies (*P* = 0.39, *I^2^* = 5%). Therefore, the fixed effects model was used to get a total summary. There was no significant difference in the live birth rate between the IVIG group and the placebo group [OR = 1.43, 95%CI (0.99, 2.07), *P*=0.05] (**Figure 5**). All plots fall in the funnel figure and are almost symmetric (**Figure 6**). Therefore, no significant publication bias was identified.

#### 3.3.3 Live birth rate between the IVIG group and low molecular weight heparin plus low-dose aspirin group

**Figure 7:**
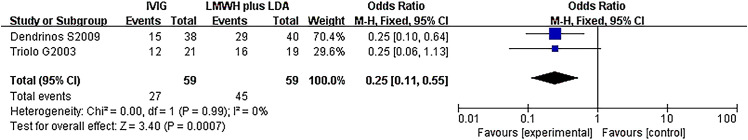
Live birth rate between the IVIG group and the LMWH plus LDA group in recurrent spontaneous abortion patients with positive for antiphospholipid antibodies (aPLs).

**Figure 8:**
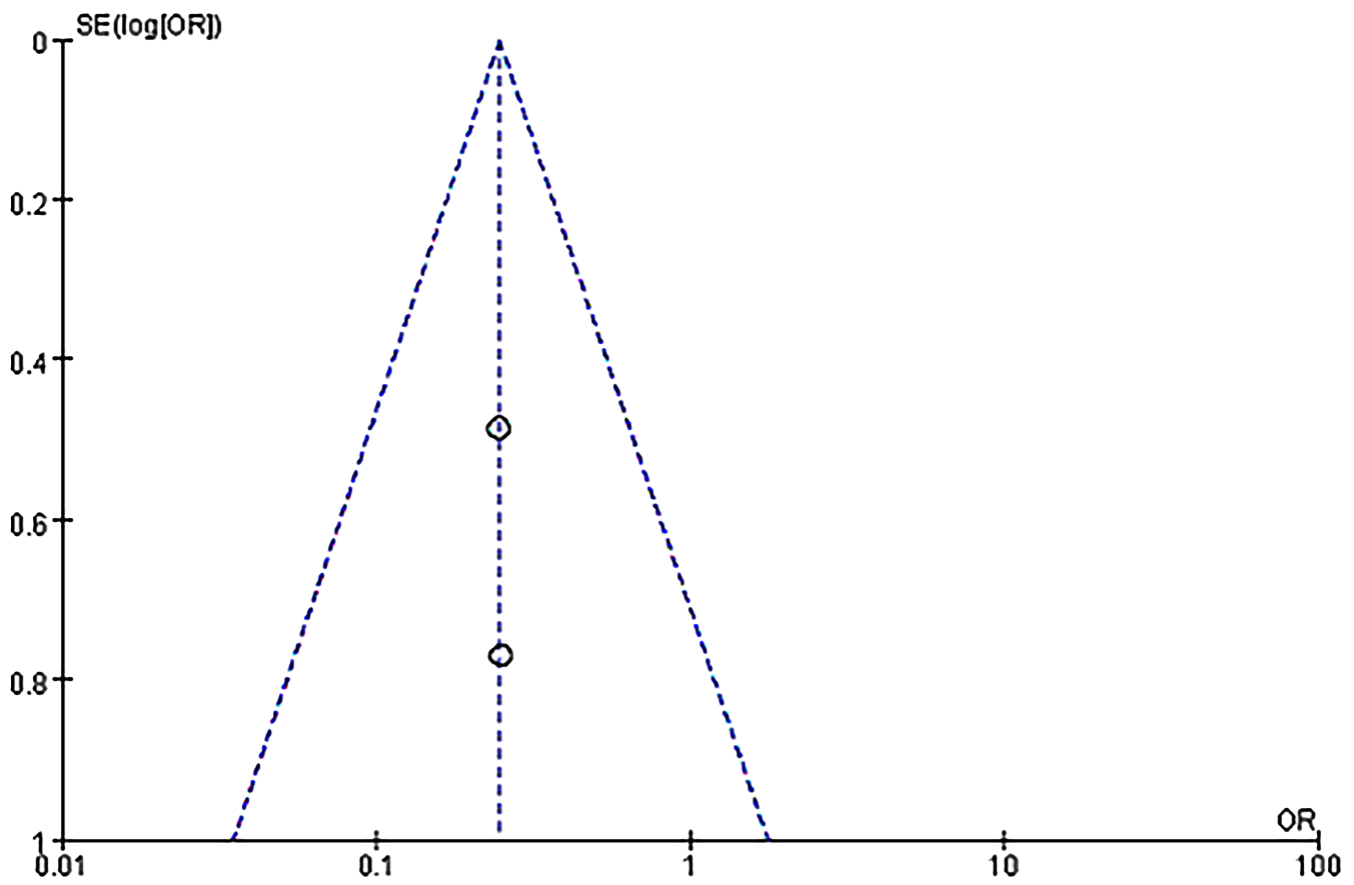
Funnel plot assessment of publication biases (OR, Odds Ratio; SE, standard error).

Another subgroup analysis was performed between IVIG treatment and LMWH plus LDA treatment. And the participants are positive for antiphospholipid antibodies (aPLs). Two studies (118 patients) reported the difference in live birth rate between IVIG treatment (59 patients) and LMWH plus LDA treatment (59 patients). There was no statistical heterogeneity among the studies (*P* = 0.99, *I^2^* = 0%). There was a significant difference in the live birth rate between the IVIG group and the LMWH plus LDA group [OR = 0.25, 95%CI (0.11, 0.55), *P*=0.0007] (**Figure 7**). All plots fall in the funnel figure and are almost symmetric (**Figure 8**). Therefore, no significant publication bias was identified.

## 4 Discussion

Although the exact mechanism of IVIG is not well elucidated, IVIG has been widely used as a non-specific immunosuppressant in the clinical treatment of many complex immune diseases, such as URSA (28). The U.S. Food and Drug Administration (FDA) has approved the use of IVIG as a first-line treatment for autoimmune thrombocytopenia, while other conditions such as RSA, antiphospholipid antibody syndrome, and repeated unexplained IVF failures remain off-label indications for this blood product (29, 30). There are no international guidelines that explicitly recommend IVIG for the routine treatment of RSA (3, 31). The effect of IVIG on URSA is controversial.

In our study, there is no significant difference between the IVIG group and the non-IVIG group [OR = 1.07, 95%CI (0.65, 1.75), *P*=0.80]. Considering that there are 5 different therapy regimens in the non-IVIG group, including LMWH plus LDA, intralipid, multivitamins, albumin, and normal saline, they may have different efficacy for URSA. This may be the reason why no statistical significance between the IVIG group and the non-IVIG group. Therefore, we performed a three-subgroup analysis according to the different therapy regimens in the non-IVIG group. Firstly, the placebo group refers to the use of no-effect reagents on URSA at the time of treatment in the non-IVIG group, including multivitamins, albumin, and saline. Secondly, the LMWH plus LDA group refers to the use of LWMH plus LDA at the time of treatment in the non-IVIG group. Thirdly, the intralipid group refers to the use of only intralipid at the time of treatment in the non-IVIG group.

The subgroup meta-analysis between the IVIG group and the placebo group suggested IVIG treatment can’t improve the live birth of URSA. Meanwhile, Hutton et al. indicated that IVIG has no positive effect on URSA in improving live birth rates, based on a systematic review and meta-analysis of 7 studies (28). However, A systematic review and meta-analysis (32) of 11 studies published in 2016 showed that IVIG can improve the live birth rate of URSA. Another systematic meta-analysis (33) of 13 RCTs published in 2022 concluded that, compared to placebo, IVIG can significantly increase the live birth rate of RSA. However, our result is inconsistent with the above two published meta-analyses (32, 33). It is worth considering whether IVIG should be used to treat URSA.

The second subgroup analysis was performed (20, 27) between the IVIG group and the LMWH plus LDA group. The result indicated the curative efficacy of the IVIG therapy group was inferior to the combination of LMWH and LDA treatment. Noteworthy, participants of two studies included in our meta-analysis were positive for aPLs. The aPLs go down as one of the most closely relevant pathogenic factors in URSA with autoimmune diseases (7). In clinical, 5% to 20% of URSA patients are positive for aPLs (7). According to the result of our subgroup analysis, IVIG was found to have a lower live birth rate compared with LDA+LMWH in our meta-analysis. However, the study conducted by Mahmoud F et al (22) showed that the IVIG group reduced the rate of preterm birth and miscarriage in people with RSA with APS, compared with placebo. Thus, the effectiveness of IVIG against URSA with positive for aPLs is controversial. It is well known the use of LMWH plus LDA has been regarded as the first-line treatment for prevention of URSA in pregnant women with positive for aPLs (34–36). Moreover, some researchers reported that IVIG can be used as a second-line treatment for URSA with positive for aPLs (36). There are currently few studies focusing on the effect of IVIG in URSA patients with positive for aPLs. Therefore, more studies are needed to show the effects of IVIG on URSA patients with positive for aPLs.

The third subgroup analysis was conducted between the IVIG group and the intralipid group. It only included one study, including 87 patients, with 48 in the IVIG group and 39 in the intralipid group, compared the effect of IVIG versus intralipid on URSA (37). The study suggested that IVIG tends to improve the live birth rate, compared to intralipid. But there was no statistical significance [OR=2.01, 95%CI (0.99,4.09)]. An article (38) published by Coulam CB in 2021 proved that intravenous infusion of intralipid significantly increases the live birth rate in patients with URSA who have increased peripheral blood NK cell activity or intimatal NK cell density. A case-series report (39) published by Plaçais L is consistent with the standpoint of the Coulam CB, which suggests an intravenous infusion of intralipid increases live birth rates and reduces miscarriage rates in patients with URSA. So intralipid can be an option for clinical trials in selected URSA patients who have failed conventional therapy and have certain immune abnormalities (elevated NK cell activity). Therefore, more studies are needed to prove the effect of intralipid on URSA in the future.

Our study had several strengths and shortcomings. Compared with the three previous systematic reviews and meta-analyses (28, 32, 33), our meta-analysis added another four RCTs (15, 20, 23, 27), and had a more rigorous assessment of the quality of the included studies, which makes our results more reliable. Furthermore, we excluded four articles (13, 40–43) that were included in the previous meta-analysis (33), whose study types were not RCTs after screening and serious discussion. Furthermore, we excluded two RCTs (44, 45) that were included in the previous meta-analysis (28, 33) because the abortion gestational age of the participants included in this RCT was after the 24^th^ week of gestation. Therefore, our meta-analysis had a smaller sample size but higher reliability. However, our findings only relate to the possibility that IVIG can improve the live birth rate of URSA patients. What is lacking is that the studies we included did not investigate the risk of adverse obstetric complications, such as premature birth, gestational hypertension, preeclampsia, eclampsia, placental abruption, and adverse fetal outcome, such as the weight and height of birth, fetal teratogenicity and so on. Due to the lack of consistency in the dose and timing of IVIG (pre-pregnancy or pregnancy) in the included RCTs, we were unable to further explore the most effective dose and optimal administration timing of IVIG for URSA.

It is important to consider whether IVIG may cause other serious complications in pregnant women and fetuses. Besides, IVIG is a blood product derived from human plasma, which theoretically carries the risk of transmitting blood-borne diseases. There have been some reports on the safety of IVIG. A study (46), that included 370 women with reproductive failures who used IVIG during their pregnancy, proved that the use of IVIG during pregnancy did not increase obstetric complications, mainly including preterm births, gestational diabetes, preeclampsia, placental abruption, placenta previa, and placenta accrete, and fetal teratogenicity. Some articles indicated that the common side effects of IVIG include headache, fever, chills, dizziness, nausea, vomiting, muscle pain, and so on (33, 46). However, these side effects often occur early before IVIG treatment. Reducing the infusion rate can alleviate the side effects (33). These will serve as available evidence for the maternal and fetal safety of IVIG use during pregnancy. Furthermore, IVIG is quite expensive, and it has been reported that a course of treatment for adults usually costs more than $10,000 (28). Because of the limited efficacy and safety of IVIG, and its high cost, whether IVIG can be used as a first-line treatment for URSA is worth exploring and considering.

## 5 Conclusion

According to our 12 high-quality, low-risk, randomized controlled trials, we may conclude that URSA patients who were defined as two or more URSA before the 24th week of gestation in our meta-analysis might not benefit from IVIG treatment compared to placebo. Besides, the treatment of LMWH plus LDA would be better than IVIG treatment in URSA patients with positive for aPLs. In the future, more RCTs are needed to explore the effect of IVIG on URSA with or without positive for aPLs. Also, more large-scale clinical trials must be conducted to investigate the optimal dosage and the best time for IVIG administration on URSA.

## Data Availability

The original contributions presented in the study are included in the article/**Supplementary Material**. Further inquiries can be directed to the corresponding authors.
