# Supplementary material for: Effect of IVIG therapy on pregnant women with unexplained recurrent spontaneous abortion: a systematic review and meta-analysis
Source: Front Endocrinol (Lausanne). 2024 Aug 14;15:1381461. doi: 10.3389/fendo.2024.1381461 (PMC11349655; doi:10.3389/fendo.2024.1381461)
Supplement: Supplementary file 1 [file Datasheet1.docx]

Supplementary Material

**Appendix S1 the systematic search strategy
1. PubMed**

#1 Abortion, Habitual OR Habitual Abortion OR Habitual Abortions OR Abortion, Recurrent OR Recurrent Abortion OR Recurrent Abortions OR Miscarriage, Recurrent OR Recurrent Miscarriage OR Recurrent Miscarriages OR Recurrent Early Pregnancy Loss

#2 Immunoglobulins, Intravenous OR Antibodies, Intravenous OR Intravenous Antibodies OR Immune Globulin, Intravenous OR Intravenous Immune Globulin OR Intravenous Immunoglobulins OR Intravenous IG OR IV Immunoglobulins OR Immunoglobulins, IV OR IVIG OR IV Immunoglobulin OR Immunoglobulin, IV OR Intravenous Immunoglobulin OR Immunoglobulin, Intravenous OR Flebogamma DIF OR Gamunex OR Globulin-N OR Globulin N OR Intraglobin OR Intraglobin F OR Intravenous Immunoglobulins, Human OR Human Intravenous Immunoglobulins OR Immunoglobulins, Human Intravenous OR Immune Globulin Intravenous (Human) OR Immunoglobulins, Intravenous, Human OR Human Intravenous Immunoglobulin OR Immunoglobulin, Human Intravenous OR Intravenous Immunoglobulin, Human OR Gammagard OR Gamimune OR Gamimmune OR Modified Immune Globulin (Anti-Echovirus Antibody) OR Privigen OR Sandoglobulin OR Venoglobulin OR Venoglobulin-I OR Venoglobulin I OR Venimmune OR Iveegam OR Alphaglobin OR Endobulin OR Gamimune N OR Gamimmune N OR Gammonativ

#1 [All Fields] AND #2 [All Fields] **the number of search outcome is 501 articles**

**2. Embase**

#1 'recurrent abortion'/exp OR 'recurrent abortion'

#2'abortion, habitual': ab,ti OR 'abortion, recurrent':ab,ti OR 'habitual abortion':ab,ti OR 'repeated abortion':ab,ti OR 'successive abortion':ab,ti OR 'recurrent abortion':ab,ti

#3 #1 OR #2

#4 'immunoglobulin'/exp OR 'immunoglobulin'

#5 'antibody protein':ab,ti OR 'endobulin':ab,ti OR 'flebogamma liquida':ab,ti OR 'gamastan':ab,ti OR 'gamimmune n':ab,ti OR 'gamma globulin':ab,ti OR 'gamimune':ab,ti OR 'gamma globulin c':ab,ti OR 'gamma immunoglobulin':ab,ti OR 'gamma globulins':ab,ti OR 'gamma-globulins':ab,ti OR 'gammagee':ab,ti OR 'gammaglobulin':ab,ti OR 'gammaglobuline':ab,ti OR 'gammimune':ab,ti OR 'gammar':ab,ti OR 'gamulin':ab,ti OR 'gamulin rh':ab,ti OR 'globuman':ab,ti OR 'glovenin i':ab,ti OR 'ig':ab,ti OR 'igam':ab,ti OR 'igc':ab,ti OR 'immune gamma globulin':ab,ti OR 'immune globin':ab,ti OR 'immune globulin':ab,ti OR 'immune globuline':ab,ti OR 'immune globulins':ab,ti OR 'immune serum globulin':ab,ti OR 'immuno':ab,ti OR 'immuno gamma globulin':ab,ti OR 'immuno globulin':ab,ti OR 'immunogammaglobulin':ab,ti OR 'immunoglobin':ab,ti OR 'immunoglobulin 17':ab,ti OR 'immunoglobulin c':ab,ti OR 'immunoglobulin c1':ab,ti OR 'immunoglobulin chain':ab,ti OR 'immunoglobulin gamma':ab,ti OR 'immunoglobulin preparation':ab,ti OR 'immunoglobulins':ab,ti OR 'immunoglobulins, intravenous':ab,ti OR 'immunoproteins':ab,ti OR 'immunoprotein':ab,ti OR 'intraglobin f':ab,ti OR 'isiven':ab,ti OR 'iveegam':ab,ti OR 'ivega':ab,ti OR 'ivig':ab,ti OR 'sandoglobin':ab,ti OR 'panglobulin':ab,ti OR 'tegelin':ab,ti OR 'tegeline':ab,ti OR 'veinoglobulin':ab,ti OR 'venoglobulin':ab,ti OR 'venoglobulin i':ab,ti OR 'venoglobulin-i':ab,ti OR 'immunoglobulin':ab,ti

#6 #4 OR #5

#7 #3 AND #6 **the number of search outcome is 975 articles.**

1. **Web Of Science**

(TS=(Abortion, Habitual OR Habitual Abortion OR Habitual Abortions OR Abortion, Recurrent OR Recurrent Abortion OR Recurrent Abortions OR Miscarriage, Recurrent OR Recurrent Miscarriage OR Recurrent Miscarriages OR Recurrent Early Pregnancy Loss)) AND TS=(Immunoglobulins, Intravenous OR Antibodies, Intravenous OR Intravenous Antibodies OR Immune Globulin, Intravenous OR Intravenous Immune Globulin OR Intravenous Immunoglobulins OR Intravenous IG OR IV Immunoglobulins OR Immunoglobulins, IV OR IVIG OR IV Immunoglobulin OR Immunoglobulin, IV OR Intravenous Immunoglobulin OR Immunoglobulin, Intravenous OR Flebogamma DIF OR Gamunex OR Globulin-N OR Globulin N OR Intraglobin OR Intraglobin F OR Intravenous Immunoglobulins, Human OR Human Intravenous Immunoglobulins OR Immunoglobulins, Human Intravenous OR Immune Globulin Intravenous (Human) OR Immunoglobulins, Intravenous, Human OR Human Intravenous Immunoglobulin OR Immunoglobulin, Human Intravenous OR Intravenous Immunoglobulin, Human OR Gammagard OR Gamimune OR Gamimmune OR Modified Immune Globulin (Anti-Echovirus Antibody) OR Privigen OR Sandoglobulin OR Venoglobulin OR Venoglobulin-I OR Venoglobulin I OR Venimmune OR Iveegam OR Alphaglobin OR Endobulin OR Gamimune N OR Gamimmune N OR Gammonativ))

**the number of search outcome is 597 articles.**

1. **Cochrane Central Register of Controlled Trials**

#1 MeSH descriptor: [Abortion, Habitual] explode all trees

#2 (Abortion, Habitual):ti,ab,kw OR (Habitual Abortion):ti,ab,kw OR (Habitual Abortions):ti,ab,kw OR (Abortion, Recurrent):ti,ab,kw OR (Recurrent Abortion):ti,ab,kw OR (Recurrent Abortions):ti,ab,kw OR (Miscarriage, Recurrent):ti,ab,kw OR (Recurrent Miscarriage):ti,ab,kw OR (Recurrent Miscarriages):ti,ab,kw OR (Recurrent Early Pregnancy Loss):ti,ab,kw

#3 #1 OR #2

#4 MeSH descriptor: [Immunoglobulins, Intravenous] explode all trees

#5 (Immunoglobulins, Intravenous):ti,ab,kw OR (Antibodies, Intravenous):ti,ab,kw OR (Intravenous Antibodies):ti,ab,kw OR (Immune Globulin, Intravenous):ti,ab,kw OR (Intravenous Immunoglobulins):ti,ab,kw OR (Intravenous Immune Globulin):ti,ab,kw OR (Intravenous IG):ti,ab,kw OR (Immunoglobulins, IV):ti,ab,kw OR (IVIG):ti,ab,kw OR (IV Immunoglobulins):ti,ab,kw OR (IV Immunoglobulin):ti,ab,kw OR (Immunoglobulin, IV):ti,ab,kw OR (Intravenous Immunoglobulin):ti,ab,kw OR (Immunoglobulin, Intravenous):ti,ab,kw OR (Flebogamma DIF):ti,ab,kw OR (Gamunex):ti,ab,kw OR (Globulin-N):ti,ab,kw OR (Globulin N):ti,ab,kw OR (Intraglobin F):ti,ab,kw OR (Intraglobin):ti,ab,kw OR (Intravenous Immunoglobulins, Human):ti,ab,kw OR (Human Intravenous Immunoglobulins):ti,ab,kw OR (Immunoglobulins, Human Intravenous):ti,ab,kw OR (Immunoglobulins, Intravenous, Human):ti,ab,kw OR (Immune Globulin Intravenous (Human)):ti,ab,kw OR (Human Intravenous Immunoglobulin):ti,ab,kw OR (Immunoglobulin, Human Intravenous):ti,ab,kw OR (Intravenous Immunoglobulin, Human):ti,ab,kw OR (Gamimune):ti,ab,kw OR (Gammagard):ti,ab,kw OR (Gamimmune):ti,ab,kw OR (Modified Immune Globulin (Anti-Echovirus Antibody)):ti,ab,kw OR (Privigen):ti,ab,kw OR (Sandoglobulin):ti,ab,kw OR (Venoglobulin-I):ti,ab,kw OR (Venoglobulin):ti,ab,kw OR (Venoglobulin I):ti,ab,kw OR (Venimmune):ti,ab,kw OR (Iveegam):ti,ab,kw OR (Alphaglobin):ti,ab,kw OR (Endobulin):ti,ab,kw OR (Gamimune N):ti,ab,kw OR (Gamimmune N):ti,ab,kw OR (Gammonativ):ti,ab,kw

#6 #4 OR #5 **the number of search outcome is 81 trials and 4 reviews.**
